# Artificial-Intelligence-Based Decision Making for Oral Potentially Malignant Disorder Diagnosis in Internet of Medical Things Environment

**DOI:** 10.3390/healthcare11010113

**Published:** 2022-12-30

**Authors:** Rana Alabdan, Abdulrahman Alruban, Anwer Mustafa Hilal, Abdelwahed Motwakel

**Affiliations:** 1 Department of Information Systems, College of Computer and Information Science, Majmaah University, Majmaah 11952, Saudi Arabia; 2 Department of Information Technology, College of Computer and Information Sciences, Majmaah University, Majmaah 11952, Saudi Arabia; 3 Department of Computer and Self Development, Preparatory Year Deanship, Prince Sattam bin Abdulaziz University, AlKharj 16278, Saudi Arabia; 4 Department of Information Systems, College of Business Administration in Hawtat bani Tamim, Prince Sattam bin Abdulaziz University, AlKharj 16278, Saudi Arabia

**Keywords:** Internet of Medical Things, oral cancer, biomedical imaging, artificial intelligence, Inception model, hybrid deep learning

## Abstract

Oral cancer is considered one of the most common cancer types in several counties. Earlier-stage identification is essential for better prognosis, treatment, and survival. To enhance precision medicine, Internet of Medical Things (IoMT) and deep learning (DL) models can be developed for automated oral cancer classification to improve detection rate and decrease cancer-specific mortality. This article focuses on the design of an optimal Inception-Deep Convolution Neural Network for Oral Potentially Malignant Disorder Detection (OIDCNN-OPMDD) technique in the IoMT environment. The presented OIDCNN-OPMDD technique mainly concentrates on identifying and classifying oral cancer by using an IoMT device-based data collection process. In this study, the feature extraction and classification process are performed using the IDCNN model, which integrates the Inception module with DCNN. To enhance the classification performance of the IDCNN model, the moth flame optimization (MFO) technique can be employed. The experimental results of the OIDCNN-OPMDD technique are investigated, and the results are inspected under specific measures. The experimental outcome pointed out the enhanced performance of the OIDCNN-OPMDD model over other DL models.

## 1. Introduction

The Internet of Medical Things (IoMT) is an extended version of the Internet of Things (IoT), which encompasses several interlinked devices that can be employed for timely support to patients and the healthcare sector [1]. Oral squamous cell carcinoma (OSCC) is a common cancer, and its existing rate seems to be increasing worldwide. Usually, the preferred therapy, primary cornerstone therapy, is a surgical treatment for OSCC [2,3]. In addition, considering the aggressive nature of OSCC, and most patients were identified with advanced locoregionally diseases, multimodality therapy and concomitant chemoradiotherapy can be imperative [4,5,6]. Instead of the above-stated treatment possibilities, the higher occurrence rate and the suboptimal treatment result form an important concern to date. The initial analysis is very important for better treatment, survival, and prognosis [7]. At the same time, a late diagnosis will hamper the quest for precision medicine in spite of the new developments in understanding the molecular system of tumors [8]. Hence, the deep machine learning (ML) method was touted to improve initial identification and decrease cancer-specific morbidity and mortality. Automatic image analysis can assist clinicians and pathologists in the initial level of OSCC and makes informed decisions regarding cancer management [9].

The dependability of automatic decisions is higher for real clinical applications [10]. Even with the promising performance, conventional deep learning (DL)-related classification lacks doctors’ capability to quantify decision uncertainty [11]. Without uncertainty measurements, physicians could not depend on decisions from DL-related automated systems in practical clinical routines. Irrespective of the robustness of a DL technique, tough diagnostic cases were unavoidable and might result in serious consequences for patients when the method is not referred for more analysis [12]. Previous methods have yet to learn that model how much confidence an individual output has. In this study, the author devises a DL oral cancer image classification structure that quantifies the output uncertainty of the methods and recommends that problematic cases with higher uncertainty values are mentioned for more analyses. This DL technique has uncertainty predictions and is compiled to assist and accelerate conventional medical workflows, not replace them [13,14]. DL techniques are frequently considered a ‘black-box’; however, the techniques are highly trustable and reliable by offering uncertain data and possibly rising overall performance [15]. In such a case, the automatic classifier is a tireless front-line clinician who requires no rest, presenting diagnoses when confident and denoting tough cases to experienced experts when uncertain.

The author in [16] applied and evaluated the efficiency of six deep convolution neural network (DCNN) methods, including the TL approach, for directly identifying pre-cancerous tongue lesions using small data of medically annotated images to identify earlier signs of OCC. DCNN method could differentiate between pre-cancerous tongue lesions and benign and distinguish five classes of tongue lesions, viz., geographic tongue, hairy tongue, fissured tongue, oral hairy leukoplakia, and strawberry tongue, with higher classifier performance. In [17], the authors developed an image classification model using the Inception-ResNet-V2 model. The authors also generated an automatic heat map to emphasize the region of the images probably to be included in the decision-making process.

Rajan et al. [18] developed a novel methodology that exploits an adapted vesselness measurement and DCNN to recognize oral cancer areas in IoT-related smart healthcare schemes. The strong vesselness filter system manages noise when preserving smaller structures. In contrast, the CNN framework significantly increases classifier performance by deblurring region of interest (ROI), which is focused on combining multi-dimension data from the feature vector selecting stage. The marked feature vector point is derived from every interconnected module in the region and applied as input to train the CNN. The author in [19] discovered the prospective application of deep learning and computer vision methods in oral cancer in the scope of images and examined the prospect of an automatic scheme for potentially recognizing oral malignant disorder having two phase channels.

Bhandari et al. [20] intend to raise the detection and classifying performances of oral tumors in a decreased processing duration. The presented method has a CNN with an adapted loss function for minimizing the fault in classifying and forecasting oral cancers by decreasing the over-fitting dataset and supporting a multiclass classifier. The presented method was tested on data samples from various data sets with four classes of oral cancers. Chan et al. [21] present a new DCNN compiled with texture mapping to identify cancerous areas and automatically mark the ROI in one method. The presented DCNN method has two collaborative branches: the lower branch performs semantic segmentation and ROI marking, whereas the upper one performs oral cancer detection. The network method will extract the tumorous regions with the upper branch and the lower one making the tumorous regions very accurate. A sliding window can be implemented for computing the texture images’ standard deviation values.

This article focuses on designing an optimal Inception-Deep Convolution Neural Network for Oral Potentially Malignant Disorder Detection (OIDCNN-OPMDD) technique. The presented OIDCNN-OPMDD technique mainly concentrates on identifying and classifying oral cancer. In this study, the feature extraction and classification process are performed using the IDCNN model, which integrates the Inception module with DCNN. To enhance the classification performance of the IDCNN model, the moth flame optimization (MFO) technique can be employed. The experimental results of the OIDCNN-OPMDD technique are investigated, and the results are inspected under various measures.

## 2. Methods

**Figure 1:**
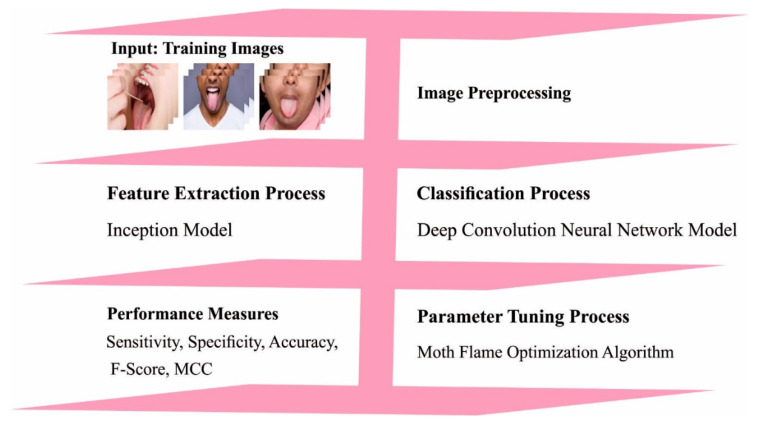
Working outline of OIDCNN-OPMDD approach.

In this article, a new OIDCNN-OPMDD technique was projected to identify and classify oral cancer in the IoMT environment. In this study, the feature extraction and classification process can be executed by using the IDCNN model, which integrates the Inception module with DCNN. To enhance the classification performance of the IDCNN method, the MFO algorithm is utilized in this study. Figure 1 depicts the overall process of the OIDCNN-OPMDD approach.

### 2.1. Pre-Processing

Firstly, this work implemented several preprocessing levels to normalize the input images. At first, the images were resized to an even size by bi-cubic interpolation on 4 × 4 neighborhood pixels. The image was resized by padlocking the sustaining quality and aspect ratio. Generally, the retinal image is yellow and has a dark background. The input image overlaps with the background image and gets eliminated to decrease noise. Matching the black contextual of the input image results in darkness as prolonged into the image details. So, pre-processing was agreed upon for eliminating the black background by fixing the pixel values for non-zero and zero to the bright zone. Then, the application of threshold, the abstraction of the green channel, was applied. The green channel will conserve extra retinal data, except red or blue. The performance of CLAHE, which is contrast-limited adaptive histogram equalization, arrived to enhance smaller areas and the retinal image quality. Then, the weighted Gaussian blur was used to increase image structure and reduce noise. The σ standard deviation and Gaussian function in 2D (*x*, *y*) are mathematically articulated in Equation (1).

(1) $$G\left(x,y\right)=\frac{1}{2\pi \sigma^{2}}\epsilon^{\frac{x^{2}+y^{2}}{2\sigma^{2}}}$$

### 2.2. Oral Cancer Recognition Module

In order to detect and classify oral cancer, the IDCNN model was utilized in this work. In this research, a DCNN mechanism with pre-trained Inception-v3 was developed [22]. The presented method is based on DTL, which aims at identifying the oral tumor from the input datasets. To extract features from the datasets, this study used pre-trained Inception-v3 architecture, and the classification model used DCNN. TL is a DL technique that exploits the module trained for the particular task as a primary point for model training for a related task. Typically, it is simpler and much quicker for network fine tuning with TL when compared to training a network from scratch. In this work, a DTL method-based Inception-v3 was carried out. The suggested method was applied for extracting features through its learned weight on the ImageNet datasets and CNN.

▪ Inception-v3 based DCNN method is deliberated to retrain; this technique comprises convolution, AvgPool, concat layer, maxpool, full connection layer, softmax function, and dropout.
▪ Average Pooling. It is a 2*D* function with a pooling size of (8 × 8) that reduces the computational complexity and the variance of the dataset. This layer enables the outcomes to flow toward the following layer.
▪ Convolution. A 299 × 299 × 3 input size is utilized through convolutional operation, and this layer produces the feature map by convoluting the input dataset.
▪ Maxpool. It is a 2D *max* pooling operation, decreasing the dataset’s variance and computation difficulty.
▪ Classified Result features like edges and average pooling are utilized to feature extraction.
▪ Concatenation. This layer is used for concatenating the different input blobs into an individual blob of output. It takes a tensor as the input, from which a similar kind of shape expect concatenation axes and return the output of individual tensor when concatenating every input.
▪ It is regarded as the normalization technique for minimizing the over-fitting in the ANN by overwhelming complex coadaptation from the trained dataset. Now, the dropout scale is regarded as 0.4, and robust model to execute averaging with the NN method. Furthermore, dropout represents the units’ hidden and visible sides in the NN model.
▪ Fully Connected. This is utilized for connecting each neuron from one layer to others that operate according to the traditional MLP-NN model.
▪ Softmax. This is utilized as the output function that operates correspondingly towards the *max* layer once it is a parameter to train through gradient descent. The exponential function causes an increment in the likelihood of the previous layer and correspondingly compares with other values; each output summation is equivalent to one.

Generally, a 2D plane forms different independent neurons, and the DCNN is composed of different layers with many 2D feature mapping plane models. There exist 4 primary segments of the DCNN. The initial one is the local perception that the global image does not need to be deduced through all the neurons in a neural network, and global and local data are attained by gathering local datasets. The second one is the convolution method. The convolution functionality is used to extract image features, and the convolutional kernels decrease the overall variables. The next one is weight sharing. This implies that the parameter of the related convolutional kernel was exploited for the whole image. Due to distinct locations in the image, the weight in the convolutional kernel would not be altered. Furthermore, convolutional operation weight sharing would considerably decrease the parameter of the convolutional kernels. The last one is the pooling layer, which is usually fixed in the CNN behindhand convolutional layer, employed to decrease the feature dimension of the efficiency of the preceding convolutional layer instantaneously to preserve data of the satisfactory crucial image.

To estimate the dot product of weight and the value in the input, a filter that is an array of weights was utilized in a convolution layer that slides over the input from a preceding layer. The procedure of backpropagation of error finds out such weights. Afterwards, an activation function that integrates component-wise non-linearity generates a feature map using every entry signifying a single neuron output from a small local area of the input. Then, the feature map is utilized for training a NN model.

As a filter is regarded, once the number of filters is high, it can extract additional feature maps and improve the model performance. Therefore, the relative imprints of 32-32-64, 32-32-32, 64-64-64, and 64-64-128 filters are employed to select the proper filter on the condition that computation resource and DCNN network performance were regraded on keeping the different influencing unchanged factors and distinct hierarchical architectures. Therefore, 64-64-64 was selected as the convolutional filter, which considers the performance, and each corresponding field size is 5 × 5.

For Inception-v3, the likelihood of each label k∈{1, …,K} for all the training instances is estimated as follows

(2) $$Q(k|z)=\frac{\;\exp\;\left(y_{k}\right)}{\sum_{i}^{k}\;\exp\;\left(y_{i}\right)},$$

In Equation (2), y signifies the non-normalized log probability. The distribution of ground truth over label p(k|z) was normalized; therefore, $\overset{\;}{\underset{k}{\sum }}p(k|z)=1$. For these systems, the loss was given using cross-entropy:

(3) $$C=\sum_{k=1}^{K}\;\log\;\left(q\left(k\right)\right)p\left(k\right).$$

For logits *yk*, the cross-entropy loss can be distinguishable, and thus it is employed in in-depth module gradient training, whose gradient has the simplest form of $\partial C/\partial y_{k}=q\left(k\right)-p\left(k\right)$, bounds between –1 and 1. Generally, this implies that the log probability of accurate labels can be increased after the cross-entropy is minimalized. Therefore, it produces some over-fitting problems. Inception-v3 regarded the distribution on labels with smooth variable ∈ independent of trained instances (*k*), from which the label distribution $p(k|z)=Z_{k,z}$ was interchanged using

(4) $$p^{\prime (k|z)}=\left(1-\epsilon \right)\partial_{k,z}+\epsilon \nu \left(k\right),$$

that is a combination of the original p(k|z) distribution with 1 − ϵ weights and the ν(k) fixed distribution with ϵ weight.

For a uniform distribution ν(k)=1/K, label smoothing normalization is employed so that it turns out to be

(5) $$p^{\prime (k|z)}=\left(1-\varepsilon \right)\delta_{k,z}+\frac{\varepsilon }{K}.$$

Consecutively, this is inferred as cross-entropy in the following

(6) $$H\left(p^{\prime },q\right)=-\sum_{k=1}^{K}\;\log\;\left(q\left(k\right)\right)p^{\prime \left(k\right)}=\left(1-\epsilon \right)H\left(p^{\prime },q\right)+\epsilon H\left(v,q\right).$$

Different activation features exist in the activation layer, namely softmax, sigmoid, and ReLU. The process is to integrate non-linear factors to improve the model condition; subsequently, it should be non-linear, and it is formulated by using Equation (7)

(7) $$f\left(x\right)=\frac{1}{1+e^{-x}}.$$

The activation function of ReLU can be formulated in the following:

(8) $$f\left(x\right)=\{\begin{array}{cc} 0, & x\leq 0,\\ x, & x>0. \end{array}$$

The activation function of the *softmax* layer can be formulated in Equation (9):

(9) $$f\left(x_{j}\right)=\frac{e^{x_{j}}}{\sum_{\;}^{\;}e^{x_{i}}}.$$

From the equation, *f*(*x*) indicates the activation function, and *x* denotes the activation function input. This is a non-linear function such as sigmoid or ReLU that can be employed for the element of convolution named activation function. If more than one pooling layer has been used for the feature map produced through the convolution layer, the computation perplexity of CNN can be decreased.

### 2.3. Hyperparameter Tuning Model

To enhance the classification performance of the IDCNN method, the MFO algorithm is utilized. MFO is an MH technique that mimics the behavior of moths in nature [23]. The major stages of MFO are defined below:

(10) MFO=(R, V, T), 

In Equation (10), *R* is used for randomly initializing the population of moths; the fitness value, *V*, determines the major function that moves the moth around the search space, and *T* shows a flag of the stopping condition.

In the major function (V), the moth location is upgraded using flames as follows:

(11) $$\vec{A}=S\left({\vec{A}}_{i},\;F_{j}\right),\;$$

In Equation (11), *S* denotes the spiral function, $A_{i}$ shows the *i*-th moths, and $F_{j}$ indicates the *j*-th flames and expresses in the following:

(12) $$S\left({\vec{A}}_{i},\;{\vec{F}}_{j}\right)={\vec{D}}_{i}\cdot e^{bl}\cdot \;\cos\;\left(2\pi l\right)+{\vec{F}}_{j},\;$$

(13) $$\vec{D}=\left|{\vec{F}}_{j}-{\vec{A}}_{i}\right|,\;$$

In Equation (12), *b* shows a constant to define the logarithmic spiral curve, and l∈[−1,1] is randomly produced. Define the distance of *i*-th moths to *j*-th flames.

The optimal solution exploitation degrades owing to the changing of moth location w.r.t $N_{pop}$ different locations in the problem. To resolve these issues there exists a method used to resolve these problems by offering more than one flame (Fno) as follows:

(14) $$\mathrm{Fno}=\mathrm{round}\;(N-iter_{c}\times \frac{N_{pop}-1}{iter_{\;\max\;}}),\;$$

Equation (14) $iter_{c}$ indicates the iterative number, $N_{pop}$ describes the maximal flame number, and $iter_{\;\max}$ specifies the stopping condition (the maximal iteration count). Algorithm 1 illustrates the key procedure of the MFO approach.
**Algorithm 1** Pseudocode of MFO Algorithm1: Generate early population of moths (A); 2: Compute the value of the fitness function of A;  3: while not T do 4:    Compute the number of flames based on Equation (14): 5:    FA = the value of fitness function of (A);  6:    if Loop ==1 then 7:       =sort(A);  8:       =sort(FA);  9:     else 10:       $F=sort\left(A_{c-1},A_{c}\right)$;  11:    $=sort\left(A_{c-1},\;A_{c}\right)$;  12:     end if  13:    for i=1 : n do 14:    for j=1 : n2 do 15:       Upgrade b and t 16:       Calculate D 17:       Upgrade A(i,j) by Equation (12) 18:    end for 19:    end for 20: end while 21: $y_{j}=A$ 22: Output: Optimum flames

## 3. Results and Discussion

**Figure 2:**
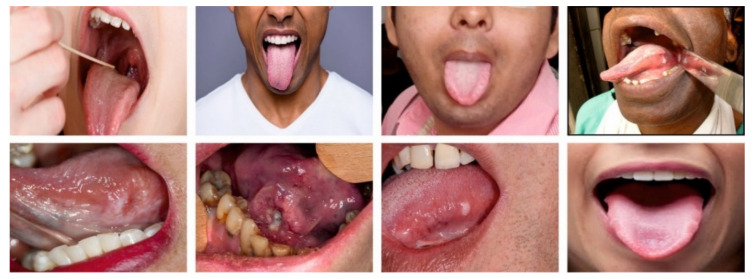
Sample images.

The oral cancer classification results of the OIDCNN-OPMDD method are investigated utilizing the oral cancer dataset from the Kaggle repository [24]. Table 1 showcases the details of the dataset. A few sample images are depicted in Figure 2. The dataset holds 131 samples with two classes. The proposed model is simulated using Python 3.6.5 tool on PC i5-8600 k, GeForce 1050 Ti 4 GB, 16 GB RAM, 250 GB SSD, and 1 TB HDD. The parameter settings are learning rate: 0.01, dropout: 0.5, batch size: 5, epoch count: 50, and activation: ReLU.

**Figure 3:**
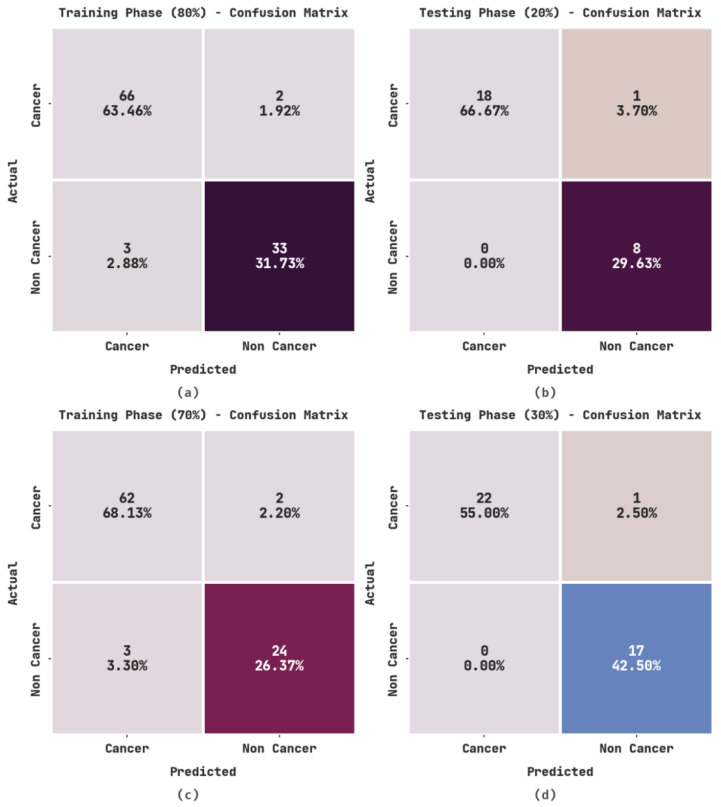
Confusion matrices of OIDCNN-OPMDD approach: (**a**) 80% of TR data, (**b**) 20% of TS data, (**c**) 70% of TR data, and (**d**) 30% of TS data.

Figure 3 illustrates the confusion matrices generated by the OIDCNN-OPMDD model. With 80% of TR data, the OIDCNN-OPMDD method categorized 66 cases into cancer and 33 into non-cancer classes. In parallel, with 20% of TS data, the OIDCNN-OPMDD algorithm categorized 18 cases into the cancer class and 8 into the non-cancer class. At the same time, with 70% of TR data, the OIDCNN-OPMDD technique categorized 62 instances into the cancer class and 24 instances into the non-cancer class. In addition, with 30% of TS data, the OIDCNN-OPMDD approach categorized 22 instances into the cancer class and 17 into the non-cancer class.

**Figure 4:**
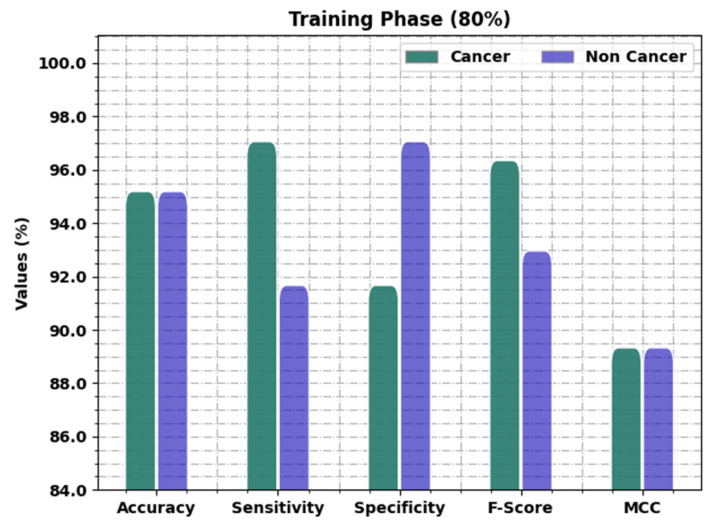
Result analysis of OIDCNN-OPMDD approach under 80% of TR data.

Table 2 and Figure 4 provide the oral cancer classification results of the OIDCNN-OPMDD model on 80% of TR data. The OIDCNN-OPMDD model identified cancer class instances with $accu_{y}$, $sens_{y}$, $spec_{y}$, $F_{score}$, and MCC of 95.19%, 97.06%, 91.67%, 96.35%, and 89.33%, respectively. In addition, the OIDCNN-OPMDD model categorized non-cancer class instances with $accu_{y}$, $sens_{y}$, $spec_{y}$, $F_{score}$, and MCC of 95.19%, 91.67%, 97.06%, 92.96%, and 89.33%, respectively. In addition, the OIDCNN-OPMDD model attained average $accu_{y}$, $sens_{y}$, $spec_{y}$, $F_{score}$, and MCC of 95.19%, 94.36%, 94.36%, 94.65%, and 89.33%, correspondingly.

**Figure 5:**
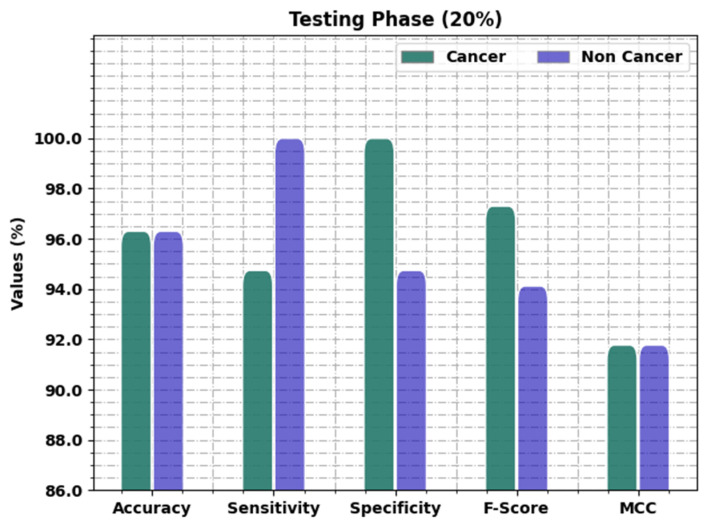
Result analysis of OIDCNN-OPMDD approach under 20% of TS data.

Table 3 and Figure 5 offer the oral cancer classification outcomes of the OIDCNN-OPMDD algorithm on 20% of TS data. The OIDCNN-OPMDD approach identified cancer class instances with $accu_{y}$, $sens_{y}$, $spec_{y}$, $F_{score}$, and MCC of 96.30%, 94.74%, 100%, 97.30%, and 91.77%, correspondingly. Moreover, the OIDCNN-OPMDD method categorized non-cancer class instances with $accu_{y}$, $sens_{y}$, $spec_{y}$, $F_{score}$, and MCC of 96.30%, 100%, 94.74%, 94.12%, and 91.77%, respectively. Further, the OIDCNN-OPMDD approach gained average $accu_{y}$, $sens_{y}$, $spec_{y}$, $F_{score}$, and MCC of 96.30%, 97.37%, 97.37%, 95.71%, and 91.77%, correspondingly.

**Figure 6:**
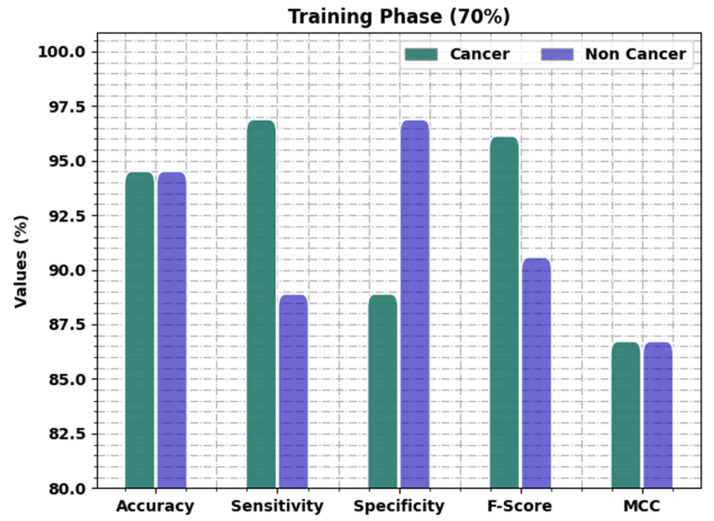
Result analysis of OIDCNN-OPMDD approach under 70% of TR data.

Table 4 and Figure 6 present the oral cancer classification results of the OIDCNN-OPMDD method on 70% of TR data. The OIDCNN-OPMDD approach identified cancer class instances with $accu_{y}$, $sens_{y}$, $spec_{y}$, $F_{score}$, and MCC of 94.51%, 96.88%, 88.89%, 96.12%, and 86.72% correspondingly. Likewise, the OIDCNN-OPMDD technique categorized non-cancer class instances with $accu_{y}$, $sens_{y}$, $spec_{y}$, $F_{score}$, and MCC of 94.51%, 88.89%, 96.88%, 90.57%, and 86.72% correspondingly. Moreover, the OIDCNN-OPMDD approach acquired average $accu_{y}$, $sens_{y}$, $spec_{y}$, $F_{score}$, and MCC of 94.51%, 92.88%, 92.88%, 93.35%, and 86.72%, correspondingly.

**Figure 7:**
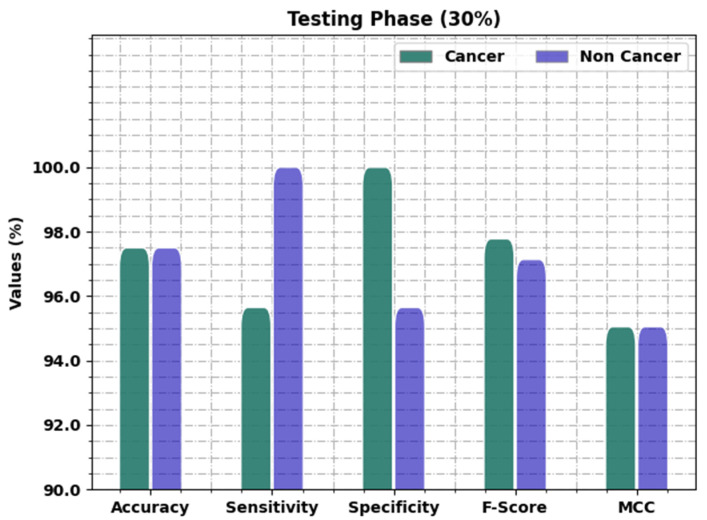
Result analysis of OIDCNN-OPMDD approach under 30% of TS data.

Table 5 and Figure 7 present the oral cancer classification results of the OIDCNN-OPMDD approach on 30% of TS data. The OIDCNN-OPMDD technique identified cancer class instances with $accu_{y}$, $sens_{y}$, $spec_{y}$, $F_{score}$, and MCC of 97.50%, 95.65%, 100%, 97.78%, and 95.05% correspondingly. Further, the OIDCNN-OPMDD approach categorized non-cancer class instances with $accu_{y}$, $sens_{y}$, $spec_{y}$, $F_{score}$, and MCC of 97.50%, 100%, 95.65%, 97.14%, and 95.05% correspondingly. Along with that, the OIDCNN-OPMDD algorithm gained average $accu_{y}$, $sens_{y}$, $spec_{y}$, $F_{score}$, and MCC of 97.50%, 97.83%, 97.83%, 97.46%, and 95.05% correspondingly.

**Figure 8:**
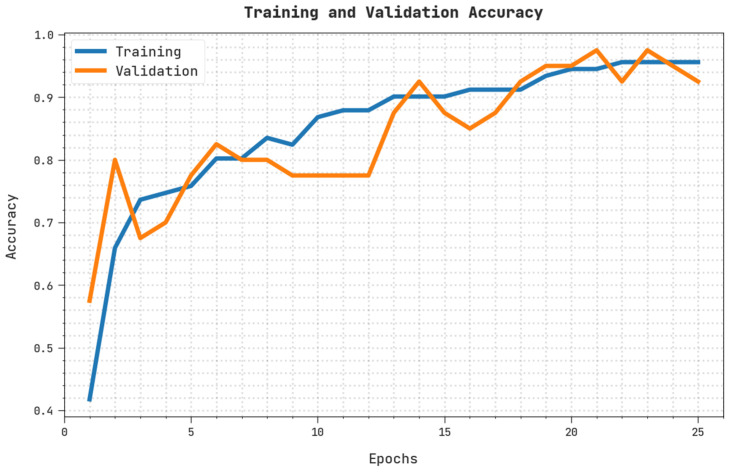
TRA and VLA analysis of OIDCNN-OPMDD approach.

The training accuracy (TRA) and validation accuracy (VLA) acquired by the OIDCNN-OPMDD approach on the test dataset is displayed in Figure 8. The experimental result inferred that the OIDCNN-OPMDD approach had achieved maximal values of TRA and VLA. The VLA is greater than TRA.

**Figure 9:**
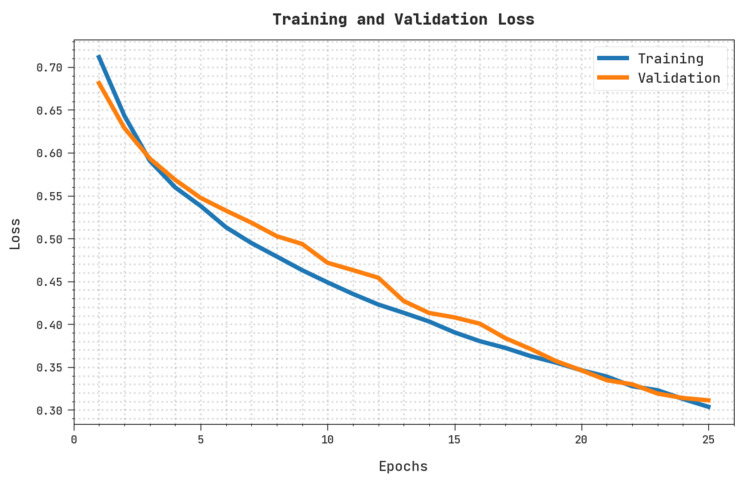
TRL and VLL analysis of OIDCNN-OPMDD approach.

The training loss (TRL) and validation loss (VLL) obtained by the OIDCNN-OPMDD technique on the test dataset are exhibited in Figure 9. The experimental result implied the OIDCNN-OPMDD method had established minimal values of TRL and VLL. Particularly, the VLL is lesser than TRL.

**Figure 10:**
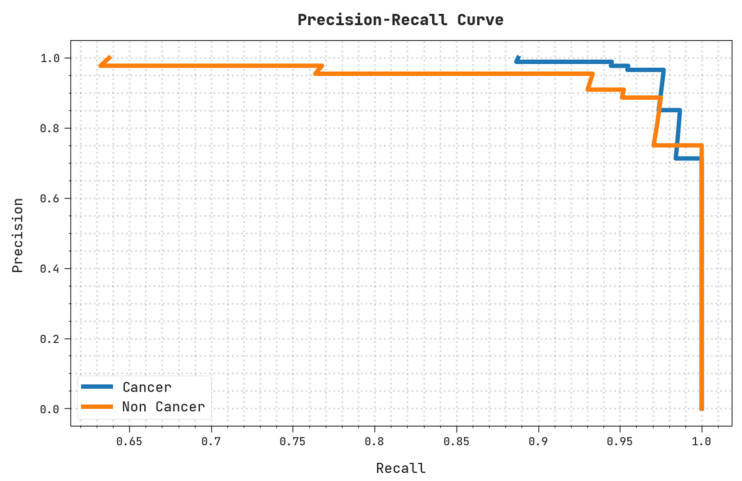
Precision–recall analysis of OIDCNN-OPMDD approach.

A clear precision–recall examination of the OIDCNN-OPMDD algorithm on the test dataset is shown in Figure 10. The figure denoted the OIDCNN-OPMDD approach has enhanced values of precision–recall values under all classes.

**Figure 11:**
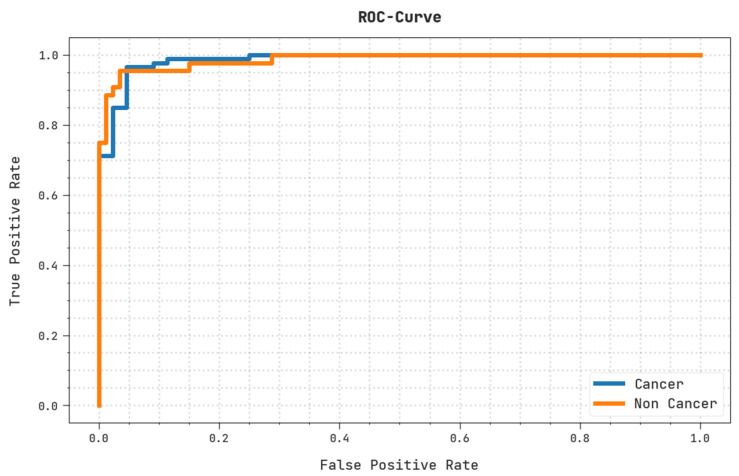
ROC analysis of OIDCNN-OPMDD approach.

A brief ROC inquiry of the OIDCNN-OPMDD technique on the test dataset is displayed in Figure 11. The outcomes denoted by the OIDCNN-OPMDD method have shown their ability to categorize distinct classes on the test dataset.

**Figure 12:**
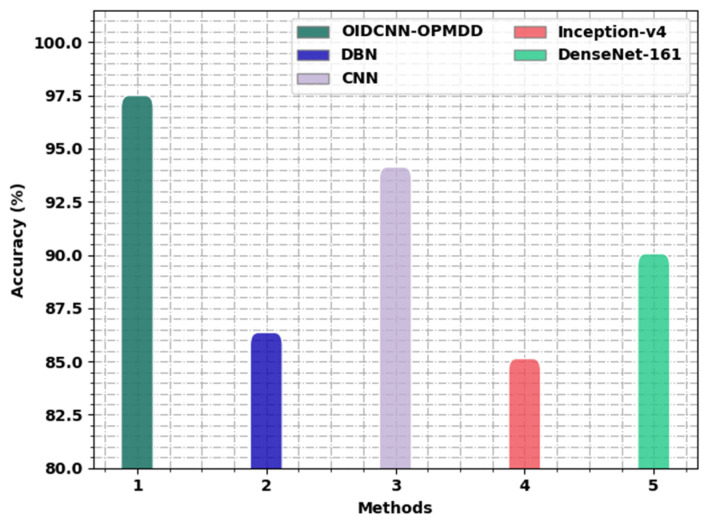
*accu_y_* analysis of the OIDCNN-OPMDD approach with existing algorithms.

Table 6 depicts detailed comparative oral classification outcomes of the OIDCNN-OPMDD model with recent DL models [10,19]. Figure 12 offers a comparative study of the OIDCNN-OPMDD model with existing models in terms of $accu_{y}$. These results indicated the ineffectual outcome of the Inception-v4 model with a minimal $accu_{y}$ of 85.14%, whereas the DBN model reported a slightly improved $accu_{y}$ of 86.36%. In addition, the DenseNet-161 method reached reasonable outcomes with an$\;accu_{y}$ of 90.06%. Next, the CNN model resulted in considerable performance with an $accu_{y}$ of 94.14%. However, the OIDCNN-OPMDD model outperformed the other ones with an increased $accu_{y}$ of 97.50%.

**Figure 13:**
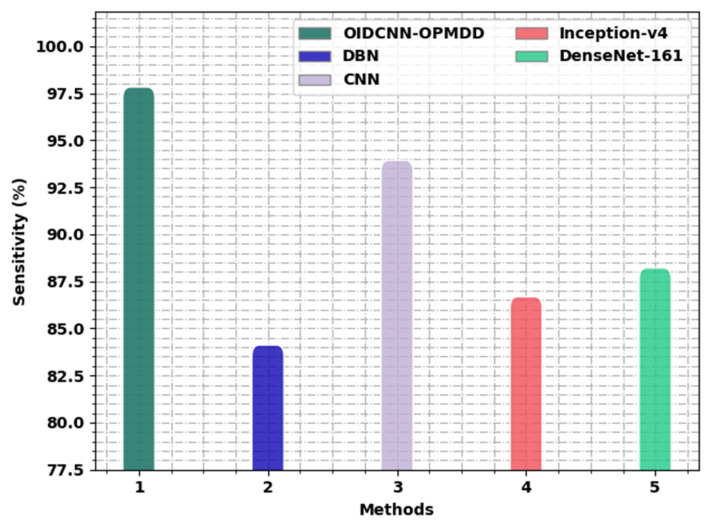
*Sens_y_* analysis of the OIDCNN-OPMDD approach with existing algorithms.

Figure 13 portrays a comparative analysis of the OIDCNN-OPMDD algorithm with existing models in terms of $sens_{y}$. These results represented the ineffectual outcome of the Inception-v4 approach with a minimal $sens_{y}$ of 86.68%, whereas the DBN method reported a slightly improved $sens_{y}$ of 84.12%. In addition, the DenseNet-161 algorithm reached reasonable outcomes with a $sens_{y}$ of 88.21%. Then, the CNN technique resulted in notable performance with a $sens_{y}$ of 93.93%. However, the OIDCNN-OPMDD approach outperformed the others with an increased $sens_{y}$ of 97.83%.

**Figure 14:**
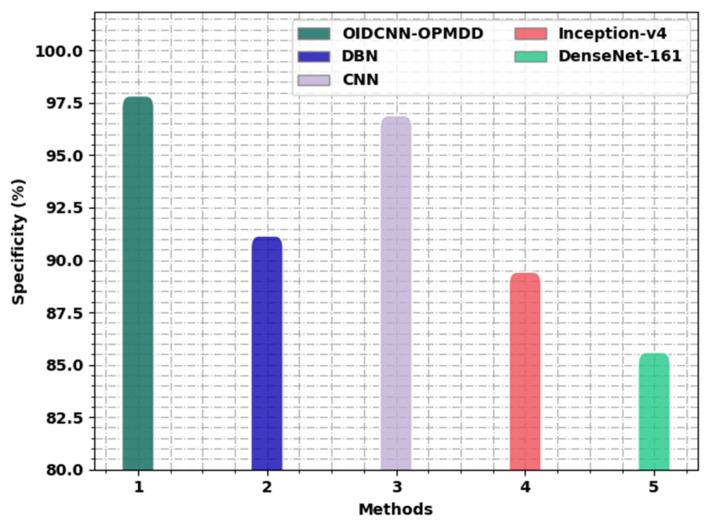
*Spec_y_* analysis of OIDCNN-OPMDD approach with existing algorithms.

Figure 14 displays the detailed study of the OIDCNN-OPMDD approach with existing algorithms in terms of $spec_{y}$. These results implicit the ineffectual outcome of the Inception-v4 technique with a minimal $spec_{y}$ of 89.42%, whereas the DBN approach managed to report a slightly improved $spec_{y}$ of 91.15%. In addition, the DenseNet-161 methodology reached reasonable outcomes with a $spec_{y}$ of 85.59%. Then, the CNN algorithm resulted in notable performance with a $spec_{y}$ of 96.89%. However, the OIDCNN-OPMDD methodology outperformed the others with an increased $spec_{y}$ of 97.83%.

**Figure 15:**
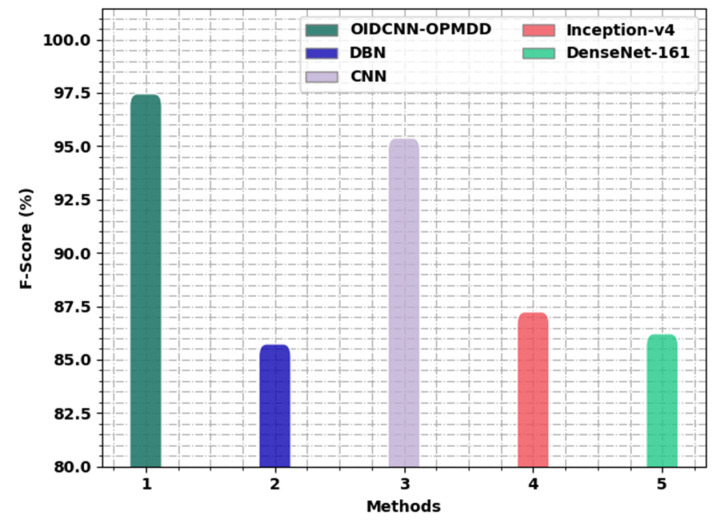
*F_score_* analysis of OIDCNN-OPMDD approach with existing algorithms.

Figure 15 exemplifies the comprehensive inception of the OIDCNN-OPMDD algorithm with existing models in terms of $F_{score}$. These results denoted the ineffectual outcome of the Inception-v4 technique with a minimal $F_{score}$ of 87.24%, whereas the DBN approach managed to report a slightly improved $F_{score}$ of 85.74%. Moreover, the DenseNet-161 methodology reached reasonable outcomes with a $F_{score}$ of 86.22%. Next, the CNN technique resulted in notable performance with a $F_{score}$ of 95.39%. However, the OIDCNN-OPMDD approach outperformed the other ones with an increased *F_score_* of 97.46%.

Thus, the OIDCNN-OPMDD model is found to be a productive solution for oral cancer detection. The enhanced performance of the proposed model is due to the optimal hyperparameter tuning using the MFO algorithm.

## 4. Conclusions

In this article, a novel OIDCNN-OPMDD approach was devised for the identification and classification of oral cancer. In this study, the feature extraction and classification process are performed using the IDCNN model, which integrates the Inception module with DCNN. To enhance the classification performance of the IDCNN method, the MFO algorithm is utilized in this study. The experimental results of the OIDCNN-OPMDD technique were investigated, and the outcomes were scrutinized under specific measures. The experimental outcome pointed out the enhanced performance of the OIDCNN-OPMDD model over other DL models. Thus, the OIDCNN-OPMDD model can be utilized for automated oral cancer recognition and classification process. In the future, the deep instance segmentation process can be combined with the OIDCNN-OPMDD model to boost the overall classification outcomes.

## Figures and Tables

**Table 1:** Dataset details.

Class	No. of Samples
Cancer	87
Non-Cancer	44
Total Number of Samples	131

**Table 2:** Result analysis of OIDCNN-OPMDD approach with distinct class labels under 80% of TR data.

Training Phase (80%)					
Labels	Accuracy	Sensitivity	Specificity	F-Score	MCC
Cancer	95.19	97.06	91.67	96.35	89.33
Non-Cancer	95.19	91.67	97.06	92.96	89.33
Average	95.19	94.36	94.36	94.65	89.33

**Table 3:** Result analysis of OIDCNN-OPMDD approach with distinct class labels under 20% of TS data.

Testing Phase (20%)					
Labels	Accuracy	Sensitivity	Specificity	F-Score	MCC
Cancer	96.30	94.74	100.00	97.30	91.77
Non-Cancer	96.30	100.00	94.74	94.12	91.77
Average	96.30	97.37	97.37	95.71	91.77

**Table 4:** Result analysis of OIDCNN-OPMDD approach with distinct class labels under 70% of TR data.

Training Phase (70%)					
Labels	Accuracy	Sensitivity	Specificity	F-Score	MCC
Cancer	94.51	96.88	88.89	96.12	86.72
Non-Cancer	94.51	88.89	96.88	90.57	86.72
Average	94.51	92.88	92.88	93.35	86.72

**Table 5:** Result analysis of OIDCNN-OPMDD approach with distinct class labels under 30% of TS data.

Testing Phase (30%)					
Labels	Accuracy	Sensitivity	Specificity	F-Score	MCC
Cancer	97.50	95.65	100.00	97.78	95.05
Non-Cancer	97.50	100.00	95.65	97.14	95.05
Average	97.50	97.83	97.83	97.46	95.05

**Table 6:** Comparative analysis of OIDCNN-OPMDD approach with existing algorithms [10,19].

Methods	Accuracy	Sensitivity	Specificity	F-Score
OIDCNN-OPMDD	97.50	97.83	97.83	97.46
DBN	86.36	84.12	91.15	85.74
CNN	94.14	93.93	96.89	95.39
Inception-v4	85.14	86.68	89.42	87.24
DenseNet-161	90.06	88.21	85.59	86.22

## Data Availability

Data sharing is not applicable to this article as no datasets were generated during the current study.
